# Molecular Epidemiology of Noninvasive and Invasive Group A Streptococcal Infections in Cape Town

**DOI:** 10.1128/mSphere.00421-19

**Published:** 2019-10-30

**Authors:** D. D. Barth, P. Naicker, K. Engel, B. Muhamed, W. Basera, B. M. Mayosi, J. B. Dale, M. E. Engel

**Affiliations:** a Department of Medicine, Faculty of Health Sciences, University of Cape Town & Groote Schuur Hospital, Cape Town, South Africa; b Wesfarmer’s Centre for Vaccines and Infectious Diseases, Telethon Kids Institute, Nedlands, Perth, Australia; c Faculty of Health and Medical Sciences, University of Western Australia, Nedlands, Perth, Australia; d National Health Laboratory Service, Groote Schuur Hospital, Cape Town, South Africa; e Division of Medical Microbiology, University of Cape Town, Cape Town, South Africa; f Hatter Institute for Cardiovascular Diseases Research in Africa, Department of Medicine, University of Cape Town, Cape Town, South Africa; g Division of Infectious Diseases, Department of Medicine, University of Tennessee Health Science Center, Memphis, Tennessee, USA; University of Maryland School of Medicine

**Keywords:** group A streptococcus, invasive GAS, molecular epidemiology, sub-Saharan Africa, vaccines

## Abstract

Group A streptococcus (GAS) is responsible for a wide range of noninvasive group A streptococcal (non-*i*GAS) and invasive group A streptococcal (*i*GAS) infections. Information about the *emm* type variants of the M protein causing GAS disease is important to assess potential vaccine coverage of a 30-valent vaccine under development, particularly with respect to how they compare and contrast with non*-i*GAS isolates, especially in regions with a high burden of GAS. We conducted a prospective passive surveillance study of samples from patients attending public health facilities in Cape Town, South Africa. We documented demographic data and clinical presentation. *emm* typing was conducted using CDC protocols. GAS was commonly isolated from pus swabs, blood, deep tissue, and aspirates. Clinical presentations included wound infections (20%), bacteremia (15%), abscesses (9%), and septic arthritis (8%). Forty-six different *emm* types were identified, including M76 (16%), M81 (10%), M80 (6%), M43 (6%), and M183 (6%), and the *emm* types were almost evenly distributed between non-*i*GAS and *i*GAS isolates. There was a statistically significant association with M80 in patients presenting with noninvasive abscesses. Compared to the 30-valent vaccine under development, the levels of potential vaccine coverage for non-*i*GAS and *i*GAS infection were 60% and 58%, respectively, notably lower than the coverage in developed countries; five of the most prevalent *emm* types, M76, M81, M80, M43, and M183, were not included. The *emm* types from GAS isolated from patients with invasive disease did not differ significantly from those from noninvasive disease cases. There is low coverage of the multivalent M protein vaccine in our setting, emphasizing the need to reformulate the vaccine to improve coverage in areas where the burden of disease is high.

**IMPORTANCE** The development of a vaccine for group A streptococcus (GAS) is of paramount importance given that GAS infections cause more than 500,000 deaths annually across the world. This prospective passive surveillance laboratory study evaluated the potential coverage of the M protein-based vaccine currently under development. While a number of GAS strains isolated from this sub-Sahara African study were included in the current vaccine formulation, we nevertheless report that potential vaccine coverage for GAS infection in our setting was approximately 60%, with four of the most prevalent strains not included. This research emphasizes the need to reformulate the vaccine to improve coverage in areas where the burden of disease is high.

## Abstract (precis)

The development of a vaccine for group A streptococcus (GAS) is of paramount importance given that GAS infections cause more than 500,000 deaths annually across the world. This prospective passive surveillance laboratory study evaluated the potential coverage of the M protein-based vaccine currently under development. While a number of GAS strains isolated from this sub-Sahara African study were included in the current vaccine formulation, we nevertheless report that potential vaccine coverage for GAS infection in our setting was approximately 60%, with four of the most prevalent strains not included. This research emphasizes the need to reformulate the vaccine to improve coverage in areas where the burden of disease is high.

## INTRODUCTION

The World Health Organization (WHO) ranked group A β-hemolytic streptococcus (GAS) (also known as Streptococcus pyogenes) the ninth leading single-organism cause of human mortality due to infectious diseases, with the majority of deaths attributable to invasive group A streptococcal (*i*GAS) diseases and rheumatic heart disease (1). The majority of cases occur in developing countries (2). It is believed that >600,000 cases of *i*GAS infection occur annually, with >160,000 deaths. Despite these alarming numbers, data on *i*GAS infection in developing countries are scant (2).

GAS is responsible for a wide range of noninvasive (non-*i*GAS) and *i*GAS diseases (3, 4). These diseases range from mild infections such as impetigo and pharyngitis to serious diseases such as streptococcal toxic shock syndrome and necrotizing fasciitis. Moreover, GAS may trigger autoimmune diseases, such as acute rheumatic fever (ARF) and rheumatic heart disease (RHD) and acute poststreptococcal glomerulonephritis (APSGN) (2), following repeated episodes of infection.

Primary prevention of GAS has been focused on the development of a vaccine; the most advanced being a 30-valent vaccine formulation (5). The GAS M protein, encoded by the *emm* gene, consists of four structural repeat blocks that have been intensively explored in epidemiological studies of GAS (6). The M serotypes in the current vaccine formulation were included on the basis of data from the developed world, with cross-coverage of certain *emm* types being observed. Information about the *emm* types causing *i*GAS disease is crucially important to assess potential vaccine coverage, especially in regions such as sub-Saharan Africa, where the burden of *i*GAS disease is among the highest (2).

There is a dearth of *emm* type data in sub-Saharan Africa; three studies (7–9) have reported the molecular typing of non-*i*GAS, and a single study (10) reported on the molecular epidemiology of *i*GAS. The African GAS infection registry (the AFRO*Strep* Study) was established to collect epidemiological data on GAS in Africa, where surveillance information is largely lacking (11). Launched in 2016 with a pilot project in South Africa, AFRO*Strep* aimed to provide an understanding of GAS disease in Africa.

By means of a prospective surveillance laboratory study, under the auspices of AFRO*Strep*, we sought to determine the extent of GAS infections and the molecular characteristics of the GAS isolates that cause disease among inpatients and outpatients attending Groote Schuur Hospital (GSH) in Cape Town, so as to inform the development of M protein-based vaccines. Additionally, we investigated how *i*GAS isolates compare and contrast with non*-i*GAS isolates with respect to their respective molecular characteristics.

## RESULTS

From February 2016 to March 2017, 488 laboratory-confirmed GAS cases were identified at the National Health Laboratory Service (NHLS) based at GSH in Cape Town. Characteristics of patients with non-*i*GAS and *i*GAS infection are listed in Table 1. The median age was 31 years (interquartile range [IQR], 21 to 45 years). GAS was more commonly isolated from males (63%). *i*GAS accounted for 46% of GAS cases. Patients with *i*GAS infection were older, with a median age of 36 years (IQR, 22 to 53 years), than patients who had non-*i*GAS infection, with a median age of 29 years (IQR, 20 to 40 years). The proportion of patients with *i*GAS infections was higher for newborns and patients ≥65 years old than for the other patients.

**TABLE 1:** Gender and age distribution of cases with noninvasive and invasive GAS infection in Cape Town^*a*^

Parameter	No. (%) of cases		
	Non-iGAS (*n* = 262)	iGAS (*n* = 226)	Total (*n* = 488)
Sex			
Female	100 (38)	76 (34)	176 (36)
Male	162 (62)	143 (63)	305 (63)
NS		7 (3)	7 (1)

Age			
≤12 mo	4 (2)	10 (4)	14 (3)
>1–5 yrs	20 (8)	12 (5)	32 (7)
>5–12 yrs	25 (10)	11 (5)	36 (7)
>12–18 yrs	12 (5)	6 (3)	18 (4)
>18–64 yrs	192 (72)	140 (62)	332 (68)
≥65 yrs	9 (3)	26 (12)	45 (9)
Unknown		21 (9)	21 (2)

a Non-*i*GAS, noninvasive group A streptococcus; *i*GAS, invasive group A streptococcus; NS, not stated.

Clinical information was available for 460 (94%) isolates (Table 2). Among non-*i*GAS cases, the most common clinical manifestations were wound infections (34%), abscesses (11%), and hand sepsis (11%). For *i*GAS infections, the most common clinical presentations were bacteremia (33%), septic arthritis (18%), and abscesses (7%). *emm* 80 was significantly associated with patients presenting with non-*i*GAS abscesses (*P* = 0.007).

**TABLE 2:** Clinical manifestations of noninvasive and invasive GAS infection by age category^*a*^

Parameter	No. of patients with clinical manifestations by age category						Total no. (%)
	≤12 mo	1–5 yrs	6–12 yrs	13–18 yrs	19–64 yrs	≥65 yrs	
Noninvasive GAS infection (*n* = 262)							
Wound infection	1	6	11	5	63	3	89 (34)
Abscess	1	4	2	2	19	1	29 (11)
Hand sepsis^*b*^	0	2	4	2	20	0	28 (11)
Hand infection	0	0	1	1	15	0	18 (6)
Lower limb infection	0	1	0	0	15	2	18 (7)
Other^*c*^	2	6	6	0	42	2	58 (26)
NS							2222 (8)

Invasive GAS infection (*n* = 226)							
Bacteremia	7	5	2	1	41	18	74 (33)
Septic arthritis	0	3	3	1	29	5	41 (18)
Abscess	2	0	1	0	13	0	16 (7)
Necrotizing fasciitis	0	0	0	1	10	1	12 (5)
Wound infection	0	0	0	0	7	1	8 (4)
Cellulitis	0	1	0	0	4	0	5 (2)
Osteomyelitis	0	0	1	0	4	0	5 (2)
Erysipelas	0	0	1	0	3	0	4 (2)
Other^*c*^	1	2	3	2	25	1	34 (15)
NS							6 (3)

Missing age data							21 (9)

a GAS, group A streptococcus; NS, not stated; N, number of cases with clinical manifestations.

b Hand sepsis is considered noninvasive because infection was inoculated through the skin.

c Other, symptoms of another disease(s) occurring in <5 patients, including osteitis, osteomyelitis, empyema, and meningitis, among others.

Information on the site of sampling was available for 475 isolates (97%); data were recorded as detailed on the laboratory requisition form. In addition to those listed in Table 3, bone, nasal swabs and tissue samples were included under “other.” Thirteen isolates had no site of isolation information; however, classifications into non-*i*GAS and *i*GAS infections were based on clinical data and additional information recorded in the notes section of the case report form (CRF).

**TABLE 3:** Sample sources of cases with noninvasive and invasive GAS infection in Cape Town^*a*^

Sample source	No. (%) of cases
Pus swab	258 (53)
Blood	90 (18)
Deep tissue	47 (10)
Abscess	36 (7)
Aspirate	31 (6)
CSF	5 (1)
Other	8 (2)
NS	13 (3)

Total	488 (100)

a CSF, cerebrospinal fluid; NS, not stated.

### Distribution of M types.

**FIG 1:**
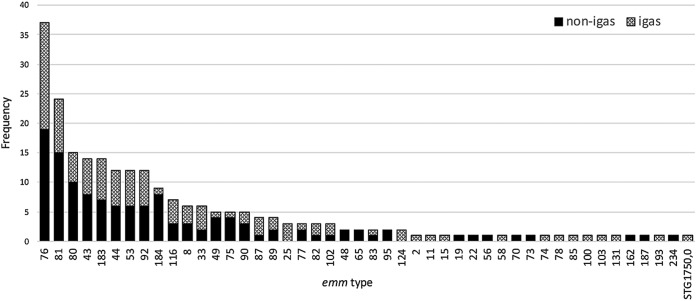
Distribution of *emm* types identified by analysis of noninvasive and invasive GAS isolates. non-igas, noninvasive group A streptococcus; igas, invasive group A streptococcus.

Molecular evaluation was conducted on 238 isolates; reasons for lack of typing included contaminated agar plates (following subculture of GAS isolates), failed PCRs, and isolates awaiting sequencing. Forty-six *emm* types were identified in 233 non-*i*GAS and *i*GAS isolates (Fig. 1). The 10 most prevalent *emm* types accounted for >67% of the isolates; these were, in descending order, M76 (16%), M81 (10%), M80 (6%), M43.7 (6%), M183.2 (6%), M44 (5%), M53 (5%), M92 (5%), M184 (4%), and M116 (3.0%). Twenty different *emm* types accounted for 86% of GAS isolates. Twenty *emm* types were represented only once, including STG1750.0, previously thought to be group G streptococcus (12). Analyses of five isolates failed to identify *emm* types, with results classified as “no hits found.”

### Vaccine coverage.

**FIG 2:**
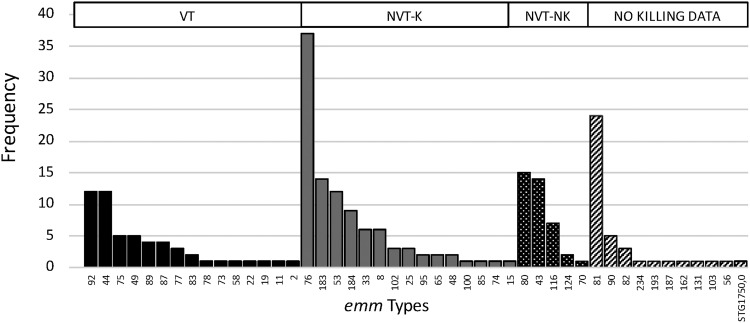
Frequency of noninvasive and invasive *emm* types observed. VT, vaccine type; NVT-K; non-vaccine type—killed; NVT-NK, non-vaccine type—not killed.

We assessed the proportion of *emm* types that were included in the 30-valent GAS vaccine currently being developed (5). Fifteen *emm* types among our cohort are included in the vaccine and were represented by 54 GAS isolates (23%) (Fig. 2). Fifteen nonvaccine *emm* types representing 100 isolates (43%) have shown cross-protection, demonstrating >50% bactericidal killing in the presence of rabbit antisera generated after vaccination with the 30-valent vaccine (5). The *emm* type (M76) most commonly identified by us is not included in the 30-valent vaccine but is among the *emm* types that evoked bactericidal antibodies. Of 233 GAS isolates, 54 were vaccine types (VT) and 100 were non-vaccine types, indicating cross coverage (identified in the figures as “NVT-K” [non-vaccine type—killed]). No information regarding potential vaccine coverage was available for 40 (17%) isolates (“No killing data”). This vaccine could cover 65% of *emm* types, corresponding to 66% of GAS cases in our setting.

### Non-*i*GAS.

**FIG 3:**
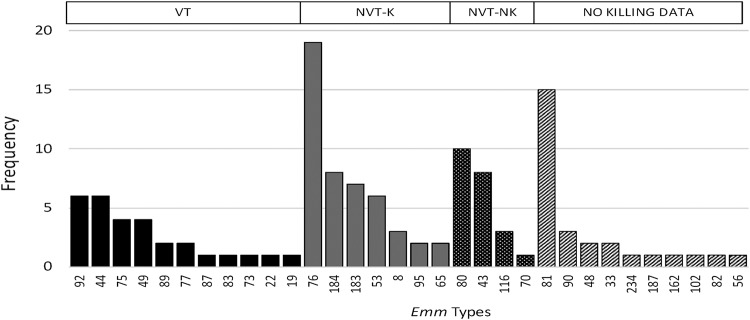
Frequency of *emm* types recovered from noninvasive GAS isolates. VT, vaccine type; NVT-K; non-vaccine type—killed; NVT-NK, non-vaccine type—not killed.

A total of 32 *emm* types were identified in 126 non-*i*GAS isolates (Fig. 3). Of these, the most prevalent *emm* types, with a frequency of >3% in the population, were M76 (15%), M81 (12%), M80 (8%), M43.7 (6%), M184 (6%), M183.2 (6%), M44 (5%), M53 (5%), M92 (5%), and M49 (3%). The 10 most prevalent *emm* types accounted for 71% of the isolates; 20 different *emm* types accounted for 90% of non-*i*GAS isolates. No new *emm* types were observed. Twelve *emm* types were presented only once.

A total of 29 (23%) non-*i*GAS *emm* types are included in the 30-valent vaccine, representing 11 different *emm* types. An additional 47 non-*i*GAS isolates (37%), representing 7 *emm* types, were included among the cross-protection isolates. The most commonly isolated *emm* type for non-*i*GAS infection, M76, was not included in the 30-valent vaccine. The potential coverage for non-*i*GAS infection in our setting is 60%. No information regarding potential vaccine coverage (“No killing data”) was available for 28 (22%) isolates.

### *i*GAS.

**FIG 4:**
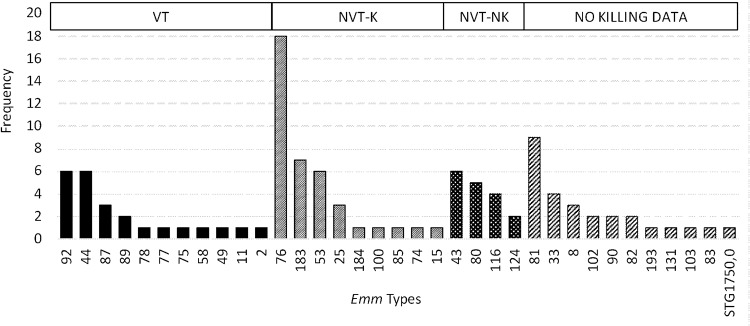
Frequency of invasive *emm* types observed. VT, vaccine type; NVT-K; non-vaccine type—killed; NVT-NK, non-vaccine type—not killed.

Thirty-five *emm* types were identified in 107 *i*GAS isolates (Fig. 4). Among these isolates, the most prevalent *emm* types, i.e., those with a frequency of >3% in the population, were M76 (17%), M81 (8%), M183.2 (7%), M43.7 (6%), M44 (6%), M53 (6%), M92 (6%), M80 (5%), M116.1 (4%), M33 (4%), and M8 (3%). The 10 most prevalent *emm* types accounted for 66% of the isolates; 20 different *emm* types accounted for 84% of the GAS cases isolated. STG1750.0 was identified in 1 isolate. Seventeen *emm* types were represented only once.

A total of 24 (22%) *i*GAS *emm* types are included in the 30-valent vaccine, representing 11 different *emm* types. An additional 39 *i*GAS isolates (36%) representing 9 more *emm* types were included among the cross-protection isolates. The most commonly isolated *emm* type for *i*GAS infection (M76) was not included in the 30-valent vaccine. The potential coverage for *i*GAS infection in our setting is 58%. No information regarding potential vaccine coverage was available for 27 (25%) isolates (“No killing data”).

### Clusters.

Among the 233 GAS isolates, we were able to assign an *emm* cluster designation (from the CDC website) to 231 isolates (Table 4) according to the cluster classification method (13). Ten *emm* type clusters were observed among the GAS isolates (Table 4). Five *emm* clusters, namely, D4, E2, E3, E6, and E4, comprised 90% of the *emm* types.

**TABLE 4:** Frequency of *emm* clusters among GAS isolates from cases of noninvasive and invasive GAS infection in Cape Town^*a*^

Cluster	Frequency	% of total
D4	59	25.54
E2	54	23.37
E3	41	17.74
E6	34	14.71
E4	20	8.65
NS	9	3.89
stG6.6	9	3.89
Clade Y	2	0.86
D2	1	0.43
E1	1	0.43
Formerly st3211.0	1	0.43

Total	231	100.00

a NS, not stated.

### Seasonal variation.

**FIG 5:**
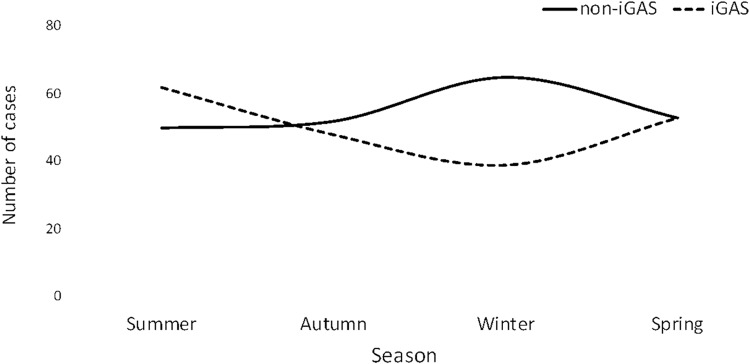
Seasonal distribution of GAS recovered from noninvasive and invasive GAS infection. non-*i*GAS, noninvasive group A streptococcus; iGAS, invasive group A streptococcus.

There was an association between the type of GAS infection and the season of the year; however, the data did not reach statistical significance (chi-square test for trend, *P* = 0.06). Non-*i*GAS infections showed a peak in the winter months. *i*GAS infections reached a trough in the winter months and peaked in the summer months (Fig. 5). Furthermore, a higher proportion of *i*GAS infections than of non-*i*GAS infections was observed during the winter months, and this difference was statistically significant (Z test, *P* = <0.001).

## DISCUSSION

This is the first report of a prospective study describing the molecular types of both noninvasive and invasive GAS infections in South Africa. The most prevalent *emm* types were almost evenly distributed between non-*i*GAS and *i*GAS isolates; a small number of *emm* types accounted for the majority of non-*i*GAS (90%) and *i*GAS (84%) cases. The proportion of *i*GAS cases was remarkably high, accounting for almost half (46%) of GAS infections in our surveillance study.

Compared with the 30-valent vaccine, only one-third of the 46 *emm* types in our study (15/46), were included, translating to levels of vaccine coverage (vaccine type and non-vaccine type killing) for non-*i*GAS and *i*GAS infection of 60% and 58%, respectively. Notably, the strains identified by us as the most prevalent, in both the non-*i*GAS and *i*GAS groups, were not included in the 30-valent vaccine. Interestingly, one *emm* type, STG1750.0, was obtained from one patient presenting with bacteremia.

We found a lower diversity of *emm* types, a result similar to others found in high-income countries (14). In our study, 20 *emm* types represented 86% of GAS isolates, which is lower than the proportion found in other studies conducted in Africa, which reported 25 *emm* types representing 70% of 70 *emm* types (8), 26 *emm* types representing 63% of 91 *emm* types for GAS pharyngitis (14), and 48 *emm* types representing 62% of 78 *emm* types for GAS skin and pharyngeal infections (9). Another study of *i*GAS isolates, conducted in Kenya, reported 88 different *emm* types (10); 74% of our strains were also found in their study. Of interest, our study findings were similar to those reported from a surveillance study conducted in Tunisia (33). Their 20 most prevalent *emm* types represented 82% of the total *emm* types, and the proportion of *i*GAS cases was 46% compared with the 49% proportion in our surveillance study. Furthermore, the *emm* types most commonly isolated in high-income countries (M1, M12, M28, M3, and M4) were not represented in our study.

Epidemiological studies have shown significant associations between *emm* type and GAS disease manifestations. *emm* types 1, 3, 5, 6, 12, 14, 17, 44, and 61 have been reported previously to be associated with superficial GAS disease (15–17), and *emm* types 1, 3, and 28 have been reported previously to be associated with *i*GAS diseases (18). In addition, *emm* types have been found to be associated with clinical manifestations, including APSGN (*emm* types 1, 4, 12, 49, 55, 57, and 60) (19) and ARF (*emm* types 1, 3, 5, 6, 11, 12, 14, 17, 18, 19, 24, 27, 29, 30, 32, and 41) (19, 20). Our study included *emm* types that have been previously shown to be associated with pharyngitis, impetigo, ARF, and PSGN.

Seasonal variations in the frequency of GAS cases have been observed in studies conducted in the United States. GAS infections have been shown to peak in the winter and early spring months and to reach a trough in the summer and autumn months (21). Similar seasonal variations have been observed in Europe (22, 23). The data regarding non-*i*GAS infections in our study are in keeping with this observation; however, for *i*GAS infection, a higher number of cases were observed in the summer months.

A new *emm* cluster typing system classifies >200 *emm* types into 48 *emm* clusters containing closely related M proteins that share structural and binding properties (24). This system predicts the M protein vaccine antigen content and serves as a framework to investigate the cross-protection phenomenon and to provide complementary hypotheses for the many variants from low-to-middle-income countries (24). Five *emm* clusters were responsible for 90% of the disease burden. It is thus conceivable that the *emm* cluster typing system could be an important typing tool to identify vaccine antigen candidates that may prove to be effective at preventing a larger proportion of GAS infections, especially in South Africa (24).

Our study had a number of limitations. (i) We were unable to assess the variation in the distribution of *emm* types over time, as reported in other studies (21, 25), since our data were collected over a one-year period. (ii) This was a hospital-based study; therefore, we could not calculate population-based incidence rates over the study period. (iii) GSH is mainly an adult hospital; hence, the number of cases in young patients was low. Therefore, caution must be applied when generalizing these findings to the lower age category. (iv) *emm* data were not available for all GAS isolated over the study period. We compared the isolates that were typed with those not typed and found no significant difference with regard to gender (chi-square test, *P* = 0.92) and non-*i*GAS and *i*GAS groups (chi-square test, *P* = 0.87). We also considered age group analysis and found no differences among patients between the ages of 13 to 18 (Z test for proportions, *P* = 0.84) and 19 to 64 years (Z test, *P* = 0.79) and those older than 64 years (Z test, *P* = 0.90). There was a difference in the younger population, among the newborns (Z test, *P* = 0.02) and those 6 to 12 years of age (Z test, *P* = 0.02). This difference could have been due to the small sample size in these age categories.

Our results have implications for current vaccine development initiatives. The current 30-valent vaccine formulation is informed by high-income countries, accounting for 90% of strains causing disease in those regions. By comparison, vaccine coverage in our study was considerably lower than the coverage in high-income countries. Even though the five most prevalent *emm* types (M76, M81, M80, M43 and M183) identified by us, accounting for 45% of our cases, are not included in the current 30-valent vaccine formulation, there is evidence of cross-protection based on detection of bactericidal antibodies that recognize shared epitopes in the N-terminal region of the *emm* types (5, 26). A small number of *emm* types are responsible for the majority of GAS cases in our setting; thus, an effective vaccine will not require diverse *emm* serotypes. Furthermore, an important finding in our study is that bactericidal activity against 33% of the non-vaccine *emm* types in our study could translate to a 43% increase in protective coverage.

The same *emm* types caused both *i*GAS and non-*i*GAS infections in our study, thus suggesting that host immune factors have a role to play in determining the severity and outcome of GAS infections in different individuals (27). Patients with serious GAS infections who present with severe clinical manifestations tend to produce elevated levels of proinflammatory cytokines in response to GAS products (28).

Although we were unable to calculate incidence rates, the proportion of *i*GAS infection at GSH was high; however, this was to be expected given that GSH is a tertiary-level hospital to which patients with severe disease are referred for care. In contrast, at a community health center, we would expect to see fewer *i*GAS infections and more non-*i*GAS infections, e.g., GAS pharyngitis.

*i*GAS infection is responsible for a substantial burden of disease, and its clinical manifestations are associated with important causes of premature mortality and morbidity. Following the first comprehensive review, published more than a decade ago, there remains a challenge in quantifying the burden of GAS disease around the world. Although more data are slowly becoming available, more work needs to be done, especially in resource-limited areas such as sub-Saharan Africa. Understanding the epidemiology and true burden of GAS diseases will help target efforts and settings in which the vaccine and other trials could be conducted. While vaccine development efforts targeting areas other than *emm* protein are under way, it must be noted that the *emm* protein vaccine is currently at the most advanced stage of development, thus warranting documentation of the corresponding variations in distributions of *emm* types. Furthermore, the reporting of *i*GAS through passive surveillance provides a platform to evaluate trends and identify new strains causing disease and, in so doing, inform the development of vaccine efforts.

## MATERIALS AND METHODS

### Study design and participants.

We conducted a prospective passive surveillance laboratory study among samples submitted from February 2016 to March 2017 to the National Health Laboratory Service (NHLS) from inpatients and outpatients attending Groote Schuur Hospital (GSH) in Cape Town. GSH is a tertiary-level hospital serving a catchment population of approximately one and a half million people (6) and forms part of a network of clinics and hospitals that are affiliated with the University of Cape Town. GSH (Groote Schuur Hospital [including state hospitals Cape Town and Western Cape, South Africa, and Groote Schuur Hospital]) provides care to more than 560,000 referrals and inpatient admissions every year, including adults (>12 years) and neonates; the NHLS also receives specimens from external primary health care clinics. We documented demographic data and clinical presentation and laboratory data from non-*i*GAS and *i*GAS infections. The study was approved by the Human Research Ethics Committee at the University of Cape Town (HREC/REF: R006/2015).

### Clinical surveillance and case definitions.

At the time of a laboratory-confirmed GAS diagnosis, a standardized case report form was completed by a study microbiologist. Clinical information was obtained by accessing the patient’s medical record. A total of 122 isolates were collected and stored at −80°C until transfer to the AFRO*Strep* laboratory.

*i*GAS was defined as GAS isolated from a sterile source such as blood, cerebrospinal fluid, or pleural fluid (29) or from a wound culture with a clinical diagnosis of necrotizing fasciitis or streptococcal toxic shock syndrome (21). GAS cultures from deep tissue (e.g., abscess) or from a biopsy sample following surgery were also considered to represent invasive infection (14). GAS isolated from a nonsterile site such as the skin or the throat was considered to be noninvasive (30).

### Molecular assays.

GAS isolates were stored in the AFRO*Strep* laboratory at −80°C in cryopreservative microbeads until DNA extraction. *emm* typing was performed as described previously (34). Briefly, isolates were subcultured on 5% sheep’s blood agar media by isolation and streaking and the plate was incubated for 24 to 48 h at 37°C in presence of 5% CO_2_. DNA was extracted using a Wizard genomic DNA purification kit per the manufacturer’s instructions, and the DNA quality and quantity were determined using a NanoDrop 1000 spectrophotometer (Thermo Scientific, Wilmington, DE, USA).

Sequencing of purified DNA was done using an ABI Prism BigDye Terminator cycle sequencing kit (Applied Biosystems, USA) at Stellenbosch University, South Africa. The sequences generated were analyzed using BioEdit v7.0.9 (Ibis Biosciences, USA). The sequences were submitted electronically to the S. pyogenes *emm* sequence database center at the CDC, which assigned all the *emm* types and subtypes (31).

### Statistics.

We evaluated the association between *emm* type and clinical symptoms using the chi-square test or Fisher’s exact test. A *P* value of <0.05 was considered to be statistically significant. All statistical analyses were performed using Stata (version 13.1; StataCorp, College Station, TX). The sample size was calculated using a prevalence of 21% for GAS pharyngitis as reported in a previous study conducted in Cape Town (32). The minimum reliable sample size was *n* = 255 to detect possible differences between non-*i*GAS and *i*GAS infection groups (95% confidence level; margin of error = 5%).
