# Erratum for Barth et al., “Molecular Epidemiology of Noninvasive and Invasive Group A Streptococcal Infections in Cape Town”

**DOI:** 10.1128/mSphere.00907-19

**Published:** 2019-12-18

**Authors:** D. D. Barth, P. Naicker, K. Engel, B. Muhamed, W. Basera, B. M. Mayosi, J. B. Dale, M. E. Engel

**Affiliations:** a Department of Medicine, Faculty of Health Sciences, University of Cape Town & Groote Schuur Hospital, Cape Town, South Africa; b Wesfarmer’s Centre for Vaccines and Infectious Diseases, Telethon Kids Institute, Nedlands, Perth, Australia; c Faculty of Health and Medical Sciences, University of Western Australia, Nedlands, Perth, Australia; d National Health Laboratory Service, Groote Schuur Hospital, Cape Town, South Africa; e Division of Medical Microbiology, University of Cape Town, Cape Town, South Africa; f Hatter Institute for Cardiovascular Diseases Research in Africa, Department of Medicine, University of Cape Town, Cape Town, South Africa; g Division of Infectious Diseases, Department of Medicine, University of Tennessee Health Science Center, Memphis, Tennessee, USA

## ERRATUM

Volume 4, no. 5, e00421-19, 2019, https://doi.org/10.1128/mSphere.00421-19. Table 2: in the last column, row 7, “2222 (8)” should read “22 (8).”

